# Women’s and men’s perspectives on breast cancer in Türkiye: strategies to improve screening participation—a cross-sectional study

**DOI:** 10.3389/fpubh.2026.1879455

**Published:** 2026-06-24

**Authors:** Burak Celik, Mehtap Manay, Ayshe Nur Yashar, Safa Toprak, Kardelen Karabulut, Sevcan Koc, Arzu Baygul Eden, Sibel Sakarya, Ece Dilege

**Affiliations:** 1 Department of General Surgery, Koç University School of Medicine, Istanbul, Türkiye; 2 Koç University School of Medicine, Istanbul, Türkiye; 3 Department of Biostatistics, Koç University School of Medicine, Istanbul, Türkiye; 4 Department of Public Health, Koç University School of Medicine, Istanbul, Türkiye

**Keywords:** breast cancer, screening, awareness, barriers, male’s attitudes, self-breast examination

## Abstract

**Background:**

Breast cancer is the most common cancer among women worldwide. Despite the availability of screening services, participation remains suboptimal in Türkiye, and evidence on the determinants of screening behaviors and men’s supportive role is limited. This study aimed to assess BC awareness among women and men, identify factors associated with screening behaviors, and explore men’s attitudes toward supporting their partners’ participation in screening.

**Methods:**

A cross-sectional survey was conducted between June 2022 and December 2024 using a convenience sampling approach among 725 participants at Koç University Hospital and affiliated institutions. The questionnaire developed by the authors based on a literature review was administered either in person or via email. Sociodemographic characteristics, health literacy, knowledge of risk factors and symptoms, screening behaviors, and self-breast examination practices were assessed through structured questionnaires.

**Results:**

Knowledge of breast cancer screening was high, with 90.5% of women recognizing that mammography enables early detection. However, among women aged ≥40 years, only 69.6% had undergone at least one mammogram. Screening uptake was significantly higher among women with a family or social history of breast cancer than among those without (70.6% vs. 29.4%, *p* = 0.002). The most frequently reported barriers to screening were the absence of symptoms (56.0%), negligence (30.3%), and difficulties accessing healthcare services (17.1%). Although 81.0% of women reported performing breast self-examination, only 22.9% did so monthly, as recommended. Among men, awareness of breast cancer symptoms was moderate, while 87.8% reported supporting their partners’ participation in screening.

**Conclusion:**

Improving access to screening services and addressing barriers such as neglect and the absence of symptoms may increase breast cancer screening uptake in Türkiye. Engaging partners and families in awareness initiatives could further support participation and facilitate earlier detection.

## 1 Introduction

Breast cancer (BC) remains the most prevalent malignancy among women worldwide. In the United States, the lifetime risk of a woman developing BC is approximately 13%, with a median age at diagnosis of 62 years (1). In Türkiye, BC similarly represents the most common cancer type among women, with one in every four female cancer diagnoses being attributed to BC. Notably, Türkiye’s median age at diagnosis is younger than that of Western countries, with a median age of 53 years (2). An analysis of Türkiye’s National Breast Cancer Database, which included the records of 20.000 patients diagnosed between 2005 and 2015, revealed that 16% of all BCs occurred in women younger than 40 years (3).

According to GLOBOCAN 2022 estimates, approximately 2.3 million women were diagnosed with BC globally, and 666,000 deaths were attributed to the disease. Based on 2020 data from the Ministry of Health Cancer Registry of Türkiye, there were 22,805 new cases, with a crude incidence rate of 54.69 per 100,000 and an age-standardized incidence rate of 43.4 per 100,000, based on the World Standard Population (4). Advances in BC survival began in the 1980s, driven by the implementation of early detection programs, increased awareness, and multimodal treatment strategies targeting invasive disease (5).

The Republic of Türkiye’s Ministry of Health offers free screening at Cancer Early Diagnosis, Screening, and Training Centers, “KETEM,” and recommends it every 2 years for individuals aged 40 and above. However, data from the Turkey Health Literacy Survey indicate that the mammography (MMG) screening rate among eligible women remains low, at 28.7%, based on 2014 data, involving 2,276 women (6). According to Eurostat data published in 2024 on EU countries, screening rates were below 40.0% in five countries, with Slovakia reporting the lowest rate at 28.5%. In contrast, Denmark, Finland, and Sweden had rates exceeding 80% (7). As a result of low screening rates in Türkiye, only 6% of BC are ductal carcinoma *in situ* at the time of diagnosis, 28.5% are stage 1, 48.3% are stage 2, and 14.5% are stage 3 (3).

Health behaviors, like self-breast examination (SBE), are recommended to promote breast awareness among women. SBE helps women become familiar with the normal look and feel of their breasts so that they can report any changes to healthcare providers quickly. SBE also introduces health-promoting habits for younger women, which may encourage them to follow up with clinical breast examinations and MMGs later in life (8). Although SBE has not been shown to increase survival rates through early detection, it is still recommended to raise awareness, especially in developing countries where access to formal screening programs may be limited (9).

While BC predominantly affects women, it is crucial to recognize that men can also develop the disease. Many individuals are unaware that men possess breast tissue and are, therefore, susceptible to BC. The American Cancer Society estimates that approximately 2,800 men in the United States will be newly diagnosed with invasive BC, and about 510 men will die from the disease in 2025 (10).

Although BC awareness and screening behaviors have been studied in various developing country contexts, most studies have focused exclusively on female participants, leaving men’s role as potential facilitators of screening largely unexplored. Furthermore, studies simultaneously assessing health literacy, symptom knowledge, risk factor awareness, and screening behaviors across both sexes remain limited. The primary aim of this study is to evaluate BC awareness across genders and sociocultural groups. In addition to assessing men’s awareness of BC, this study also aimed to evaluate their attitudes toward encouraging their partners to participate in screening programs. The findings could lay the foundation for future policies in Türkiye and developing countries aimed at enhancing the population’s acceptance, participation, and awareness of BC screening programs.

## 2 Methods

### 2.1 Study design and participants

A cross-sectional survey was conducted by our medical students who joined this study, from June 2022 to December 2024, among women and men presenting to outpatient clinics, either as patients or as accompanying companions at Koç University Hospital across multiple departments, excluding the Breast and Gynecology Clinics, where women are already more aware and have their screening, which could cause bias. Koç University employees, students, and Koç Holding employees were also eligible to participate. Medical students and hospital staff were not included in the study. Exclusion criteria included a diagnosis of BC, age below 18, and inability to read or write in Turkish. Participants completed a questionnaire, either in person on paper or via email using Qualtrics (Qualtrics, Provo, UT, USA). Access to the survey data was restricted to the research team through password-protected accounts with role-based access controls. Higher education encompasses bachelor’s, master’s, and/or doctoral programs, while pre-university education includes primary, middle school, and/or high school education. Ultimately, OpenEpi software was used to determine the sample size, based on a 25% frequency of MMG utilization, a 3% margin of error, and a 95% confidence level, resulting in a minimum required sample size of 520 (11). The study received approval from the Koç University Institutional Review Board (2022. 359. IRB3.157).

### 2.2 Study instrument

A questionnaire was carefully designed for this study, drawing upon validated instruments from previous research (8, 12–16). Two separate versions of the questionnaire were prepared for men and women, covering sociodemographic characteristics, health literacy level, knowledge of BC risk factors and warning signs, and attitudes toward BC screening and SBE practices.

Each questionnaire was divided into five main domains. Domain 1 encompasses health literacy, as measured by the short form of the Health Literacy Scale (17, 18). Domain 2 evaluates knowledge of BC risk factors and warning signs. Domain 3 examines SBE practices. Domain 4 addresses various aspects of BC screening, and Domain 5 assesses overall knowledge related to BC (Supplementary Questionnaire 1).

Men’s BC questionnaire additionally covered the attitudes toward their partner’s breast cancer screening practices found in Domain 3 (Supplementary Questionnaire 2).

### 2.3 Data collection and statistical analysis

A total of 725 respondents participated in this study. All data collected were reviewed, coded, and computerized. Descriptive statistics, including mean, standard deviation, median, minimum, and maximum, were used for quantitative variables. Qualitative variables were presented as frequencies and percentages. SPSS 26 (Statistical Package for the Social Sciences) software was used for statistical analysis. Continuous variables were assessed for normality using the Kolmogorov–Smirnov test. Normally distributed variables were compared using the independent samples *t*-test; non-normally distributed variables were compared using the Mann–Whitney U test. One-way ANOVA was used to compare mean health literacy scores across barrier subgroups. Categorical variables were analyzed using the chi-square test or Fisher’s exact test, as appropriate. Binary logistic regression analysis was performed to identify independent predictors of MMG uptake among women aged 40 and above. The dependent variable was MMG uptake (yes/no), and independent variables included age, education level, monthly income, health literacy score, family and social history of BC, occupation, marital status, and health insurance type. Results are reported as odds ratios (OR) with 95% confidence intervals (CI). Model fit was assessed using the Hosmer-Lemeshow goodness-of-fit test and Nagelkerke R^2^ Factors associated with the outcome were examined using multivariable binary logistic regression. A *p*-value of <0,05 was considered statistically significant. The following formula evaluates the health literacy scale (Index = (Mean — 1) × 50/3). The mean is calculated by dividing the total score of the scale by the number of items. The index value, calculated using the formula, ranges from 0 to 50, with higher scores indicating better health literacy. The scale consists of 11 items and uses a 4-point Likert-type response format ranging from 1 (very difficult) to 4 (very easy) (Supplementary Table 1).

## 3 Results

### 3.1 Demographic characteristics

A total of 538 women and 187 men participated in this study, and the demographic characteristics are summarized in Table 1. The mean age of the women participants was 40.68 ± 13.04 years, ranging from 18 to 81 years, with 293 (54.4%) women being 40 years or older. For male participants, the mean age was 40.38 ± 12.39 years, with a range of 18 to 70 years. Most women (61.15%) and men (66.8%) were reported as married. Regarding education level, most women (56.51%) had at least a college degree, whereas men were more likely to have pre-university education (49.7%). Based on health insurance status, 91.26% of women and 95.2% of men reported having both public and private insurance. Concerning monthly income, 34.41% of women and 13.3% of men reported earnings below or at minimum wage, while 68.59 and 86.7% reported earnings above the minimum wage, respectively. The mean health literacy score of the women and men participants was 34,386 ± 9,036 and 34,244 ± 9,327 out of 50 (maximum score), respectively.

**Table 1:** Demographic characteristics of the participants.

Demographic characteristics	Women (n:538)	Men (n:187)	Total (n:725)
Age			
(Mean ± Sd)	40.68 ± 13.04	40.38 ± 12.39	40.61 ± 12.87
Minimum-Maximum	18–81	18–70	18–81
40 and Above 40	293 (54.4%)	-	-
Minimum-Maximum	40–81	-	-
Marital status			
Single	209 (38.85%)	62 (33.2%)	271 (37.38%)
Married	329 (61.15%)	125 (66.8%)	454 (62.62%)
Level of education			
Pre-university	234 (43.49%)	93 (49.73%)	327 (45.10%)
Higher education	304 (56.51%)	94 (50.27%)	398 (54.90%)
Health insurance			
Public	326 (60.59%)	98 (52.4%)	424 (58.48%)
Private	24 (4.46%)	14 (7.5%)	38 (5.24%)
Both	141 (26.21%)	66 (35.3%)	207 (28.55%)
None	47 (8.74%)	9 (4.8%)	56 (7.72%)
Monthly income			
Below minimum wage	169 (31.41%)	25 (13.3%)	194 (26.76%)
Above minimum wage	369 (68.59%)	162 (86.7%)	531 (73.24%)
Health literacy score			
Mean ± Sd	34.386 ± 9.036	34.244 ± 9.327	34.302 ± 9.188

Sd, standart deviation.

### 3.2 Breast cancer background and health care behaviors

**Figure 1:**
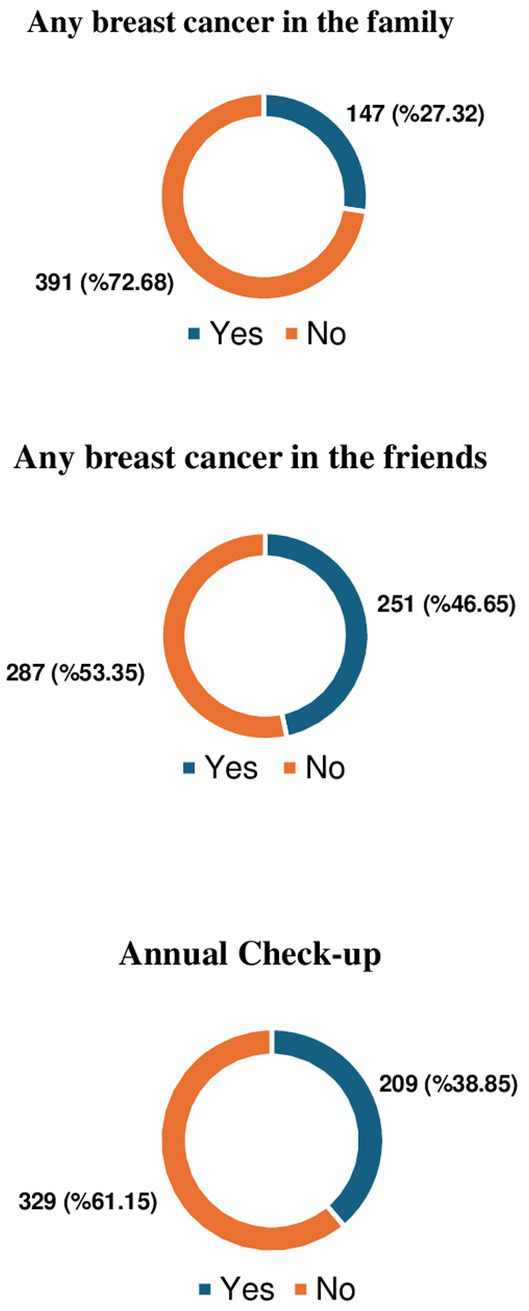
Breast cancer background and health care behaviors of women.

One hundred and forty-seven (27.32%) of women and 31 (16.6%) of men had a family history of BC (Figure 1). Two hundred fifty-one women (46.65%) and 37 men (19.8%) reported having friends diagnosed with BC. A minority of women (24.91%) and men (11.7%) indicated regular medical supervision. Two hundred and nine (38.8%) of women and 63 (33.6%) of men had an annual check-up examination habit.

### 3.3 Knowledge of symptoms

Participants’ responses regarding their knowledge of BC symptoms are presented in Table 2. Women with higher education demonstrated significantly higher awareness of BC symptoms compared to those with pre-university education, particularly for recognizing a palpable breast lump (59.50% vs. 40.40%, *p* = 0.004), a mass under the armpit (62.39% vs. 37.61%, *p* < 0.001), breast pain (61.69% vs. 38.31% vs. *p* = 0.007), and nipple bleeding or discharge (61.94% vs. 38.06%, *p* = 0.006). Nipple retraction (61.04% vs. 38.9%, *p* = 0.049), breast swelling (62.0% vs. 38.0%, *p* = 0.017), and enlargement of the breast (66.15% vs. 33.85%, *p* = 0.011). Similarly, men with higher education showed greater symptom awareness, including palpable breast lumps (55.32% vs. 44.68%, *p* = 0.016), breast swelling (58.75% vs. 41.25%, *p* = 0.045), and bloody nipple discharge (67.35% vs. 32.65%, *p* = 0.005).

**Table 2:** All participants’ knowledge of breast cancer symptoms.

Symptoms		Women (n:538)	Level of education			Men (n:187)	Level of education		
			Pre-university	Higher education	*p*-value		Pre-university	Higher education	*p*- value
Palpable breast lump	Yes	437 (81.23%)	177 (40.50%)	260 (59.50%)	**0.004**	141 (75.40%)	63 (44.68%)	78 (55.32%)	**0.016**
	No	101 (18.77%)	57 (56.44%)	44 (43.56%)		46 (24.60%)	30 (65.22%)	16 (34.78%)	
Breast pain	Yes	295 (54.83%)	113 (38.31%)	182 (61.69%)	**0.007**	88 (47.06%)	38 (43.18%)	50 (56.82%)	0.091
	No	243 (45.17%)	121 (49.79%)	122 (50.21%)		99 (52.94%)	55 (55.56%)	44 (44.44%)	
Nipple retraction	Yes	249 (46.28%)	97 (38.96%)	152 (61.04%)	**0.049**	33 (17.65%)	15 (45.45%)	18 (54.55%)	0.588
	No	289 (53.72%)	137 (47.40%)	152 (52.60%)		154 (82.35%)	78 (50.65%)	76 (49.35%)	
Orange peel appearance (peau d’orange) in the breast	Yes	188 (34.94%)	74 (39.36%)	114 (60.64%)	0.156	29 (15.51%)	12 (41.38%)	17 (58.62%)	0.328
	No	350 (65.06%)	160 (45.71%)	190 (54.29%)		158 (84.49%)	81 (51.27%)	77 (48.73%)	
Bleeding or discharge from the nipple	Yes	289 (53.72%)	110 (38.06%)	179 (61.94%)	**0.006**	49 (26.20%)	16 (32.65%)	33 (67.35%)	**0.005**
	No	249 (46.28%)	124 (49.80%)	125 (50.20%)		138 (73.80%)	77 (55.80%)	61 (44.20%)	
Mass under the armpit	Yes	351 (65.24%)	132 (37.61%)	219 (62.39%)	**<0.001**	92 (49.20%)	42 (45.65%)	50 (54.35%)	0.272
	No	187 (34.76%)	102 (54.55%)	85 (45.45%)		95 (50.80%)	51 (53.68%)	44 (46.32%)	
Breast swelling	Yes	250 (46.47%)	95 (38%)	155 (62%)	**0.017**	80 (42.78%)	33 (41.25%)	47 (58.75%)	**0.045**
	No	288 (53.53%)	139 (48.26%)	149 (51.74%)		107 (57.22%)	60 (56.07%)	47 (43.93%)	
Redness and crusting of the breast skin	Yes	168 (31.23%)	63 (37.50%)	105 (62.50%)	0.059	29 (15.51%)	11 (37.93%)	18 (62.07%)	0.167
	No	370 (68.77%)	171 (46.22%)	199 (53.78%)		158 (84.49%)	82 (51.90%)	76 (48.10%)	
Enlargemet of the breast	Yes	130 (24.16%)	44 (33.85%)	86 (66.15%)	**0.011**	37 (19.79%)	18 (48.65%)	19 (51.35%)	0.883
	No	408 (75.84%)	190 (46.57%)	218 (53.43%)		150 (80.21%)	75 (50%)	75 (50%)	

Statistical comparisons between education levels were performed using the chi-square test. Bold values were considered statistically significant (*p*-value ≤ 0.05).

### 3.4 Knowledge of risk factors

The women participants’ knowledge of BC risk factors is presented in Supplementary Table 2. Being women (60.42% vs. 39.58%, *p* = 0.050), having a family history of BC (60.82% vs. 39.18%, *p* < 0.001), being not physically active (67.39% vs. 32.61%, *p* = 0.021), aging (73.13% vs. 26.87%, *p* = 0,003) and usage of oral contraceptive pills (64.81% vs. 35.19%, *p* = 0.051) were found to differ significantly between higher educated and pre-university educated participants, respectively. Alcohol consumption and obesity are known modifiable risk factors for BC, which are not well recognized by our study group.

### 3.5 Knowledge of breast cancer screening

BC screening recommendations in Türkiye were assessed in 538 women, stratified by education level and monthly income (Supplementary Table 3). Among the respondents, 90.52% knew that BC can be detected early through MMG, with no significant difference observed based on educational level (*p* = 0.153). However, awareness was significantly higher among women earning above the minimum wage than among those earning below it (70.23% vs. 29.77%, *p* = 0.011). 44.80% of participants identified 40 years as the recommended age for initiating screening in Türkiye. Additionally, 57.81% of women believed MMG should be performed annually, with a notable income-related difference (*p* = 0.045) but no significant variation by education (*p* = 0.592).

### 3.6 Screening behaviors

Supplementary Figure 1 examines screening behaviors among women aged 40 and above who have undergone MMG at least once (*n* = 293), stratified by education level and family or social history of BC. Binary logistic regression identified four independent predictors of MMG uptake (Table 3). Increasing age was associated with decreased odds of MMG uptake (OR 0.890, 95% CI 0.847–0.936, *p* < 0.001). Higher health literacy score was associated with increased odds of uptake (OR 0.960, 95% CI 0.931–0.991, *p* = 0.011). Having a friend or acquaintance diagnosed with BC was the strongest predictor, associated with a 2.6-fold increase in odds (OR 2.632, 95% CI 1.467–4.721, *p* = 0.001). Having both public and private health insurance was associated with lower odds compared to public insurance alone (OR 0.406, 95% CI 0.196–0.839, *p* = 0.015). The overall model was significant (χ^2^ = 63.131, *p* < 0.001; Nagelkerke R^2^ = 0.275; Hosmer-Lemeshow *p* = 0.045). Supplementary Figure 2 highlights screening location and frequency patterns among 238 women who had undergone MMG. Regarding screening location, the majority utilized public hospitals (42.44%), followed closely by private hospitals (41.60%), while a smaller proportion accessed services through “KETEM” (Cancer Early Diagnosis, Screening, and Training Center) at 11.34%. Regarding screening frequency, only 29.83% of women adhered to the recommended annual MMG schedule, while 25.63% reported undergoing MMG once or every 2 years. A notable 44.54% indicated randomly, reflecting a lack of consistent adherence to screening guidelines. These findings underscore the need for targeted interventions to improve adherence to regular screening and encourage greater utilization of dedicated cancer screening centers, such as “KETEM.”

**Table 3:** Binary logistic regression analysis—independent predictors of mammography uptake among women aged ≥40 years.

Variable	OR	95% CI (Lower)	95% CI (Upper)	*p*-value
Age	0.890	0.847	0.936	**<0.001**
Education level (higher vs. pre-university)	0.838	0.444	1.582	0.585
Monthly income (above vs. below minimum wage)	0.664	0.357	1.235	0.196
Health literacy score	0.960	0.931	0.991	**0.011**
Family history of breast cancer	1.394	0.732	2.654	0.312
Social history of breast cancer^*^	2.632	1.467	4.721	**0.001**
Occupation (overall)	—	—	—	0.391
Active working vs not working	1.309	0.642	2.670	0.459
Student vs not working	2.022	0.115	35.406	0.630
Retired vs not working	0.634	0.255	1.579	0.328
Marital status (married vs. single)	1.237	0.639	2.395	0.528
Health insurance (ref: public only)	—	—	—	0.113
Private vs public	0.776	0.178	3.379	0.736
Both public & private vs public	0.406	0.196	0.839	**0.015**
None vs public	0.675	0.167	2.734	0.582

OR, Odds ratio; CI, Confidence interval.

Bold values were considered statistically significant (*p*-value ≤ 0.05).

### 3.7 Barriers to mammography screening

**Figure 2:**
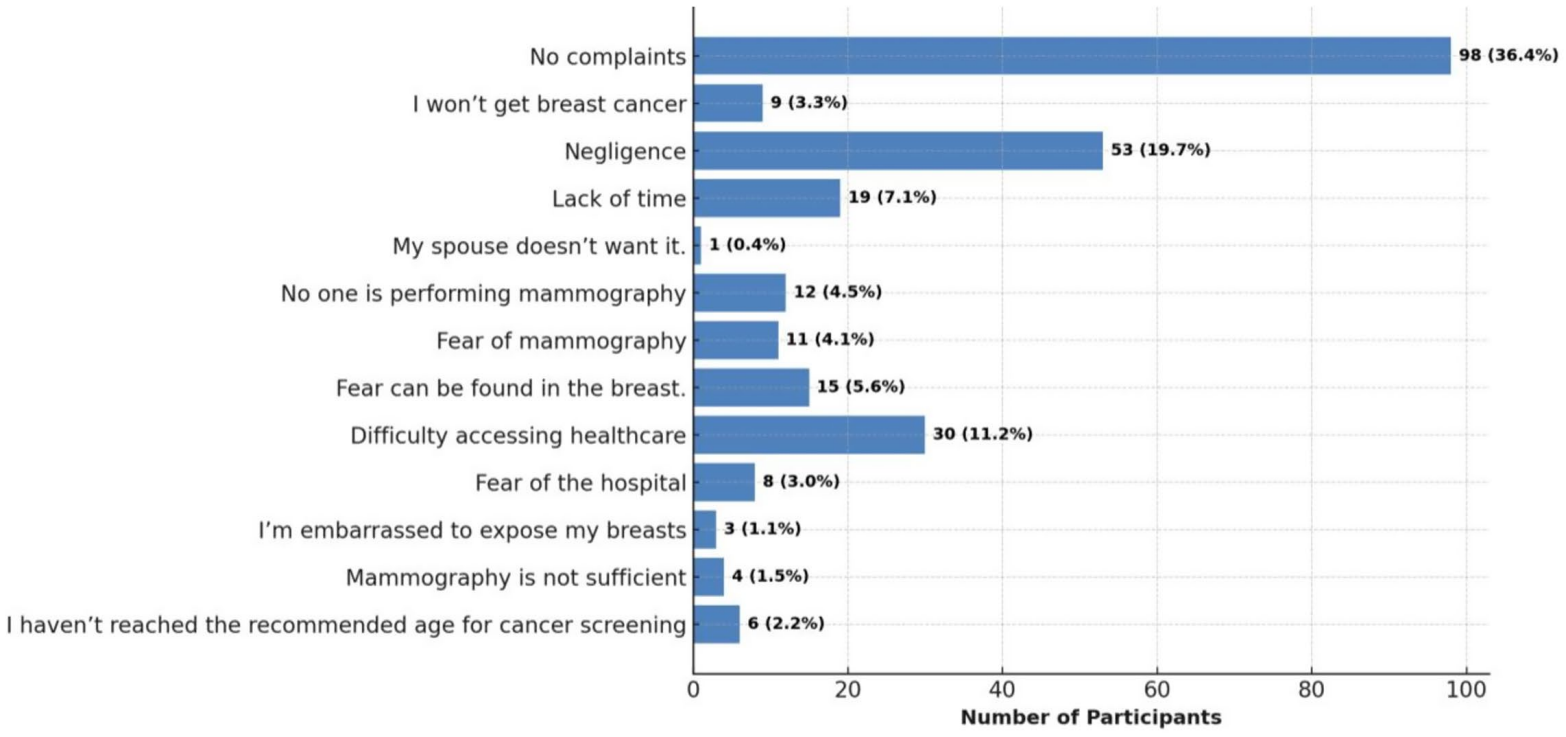
Reasons for participants who have not performed MMG before and irregular follow-up over age 40 (Participants were allowed to select more than one reason).

Figure 2 focuses on the reasons for irregular MMG routines among 175 women aged 40 and above. The most frequently cited reason was the absence of complaints, reported by 56% of participants, followed by negligence (30.29%) and difficulty accessing healthcare (17.14%). Other barriers included a lack of time (10.86%) and fear of discovering a breast abnormality (8.57%). A smaller percentage of participants reported fear of MMG (6.29%), the perception that no one is performing MMG (6.86%), and the belief that they had not yet reached the recommended age for screening (3.43%). Rarely mentioned reasons included embarrassment about exposing their breasts (1.71%), fear of hospitals (4.57%), the perception that MMG is insufficient (2.29%), and spousal disapproval (0.57%). When examining these groups by average health literacy scores, those who reported “no complaints” had the highest average score of 34.25, followed by those citing “negligence” at 31.76, “difficulty accessing healthcare” at 32.79, and “lack of time,” with the lowest score of 28.58. These findings suggest that even individuals with relatively higher health literacy may avoid regular screening due to a perceived lack of symptoms. In comparison, lower health literacy correlates with more practical barriers, such as time constraints. These findings suggest that addressing misunderstandings, increasing accessibility, and alleviating fear may be important factors in supporting regular screening attendance.

### 3.8 Source of information

The general sources of information about BC among women aged 40 and older have been examined, comparing those who have undergone MMG (*n* = 204) with those who have not (*n* = 89) (Supplementary Table 4). Family and friends were identified as sources for 44.61% of women, while healthcare workers provided information to 66.67% of women. Additionally, 34.31% received information through TV or radio, 9.31% from books, and 10.29% from newspapers. The Internet was noted as a source by 45.10% of the respondents, with specific platforms contributing as follows: Facebook (8.33%), Twitter (5.88%), YouTube (11.76%), and Instagram (15.69%). In contrast, among women who did not undergo MMG, 50.56% cited family and friends as their sources of knowledge, and 55.06% indicated healthcare workers. TV or radio was reported by 35.95% of these women, while books and newspapers accounted for 7.86 and 6.74%, respectively. The Internet served as a source for 49.44% of this group, with platform-specific awareness reported from Facebook (5.62%), Twitter (2.25%), YouTube (14.61%), and Instagram (16.85%).

### 3.9 Self-breast examination

Supplementary Table 5 examines participants’ knowledge and habits related to SBE. Among 538 women, 81.04% reported performing SBE. The frequency of SBE varied significantly by education level (*p* < 0.001). The recommended monthly SBE was reported by 22.94% of women, predominantly those with higher education (63% vs. 37%). The results highlight the importance of encouraging regular and proper SBE, especially among women with lower levels of education, to improve early detection of BC.

### 3.10 Responses to statements about breast cancer

Participants’ responses to statements about BC were analyzed to assess associations based on gender and education levels. Significant gender differences were observed across multiple statements. For example, when responding to the statement “BC is one of the rarest diseases,” women were more likely to answer correctly and disagree (78.25%) compared to men (66.84%), indicating greater awareness among women (*p* < 0.001). Similarly, for the statement “BC cannot be seen in young women,” a higher proportion of men (4.28%) than women (3.53%) agreed incorrectly (*p* = 0.005), suggesting a misconception about age-related breast cancer risk among some male participants. Another notable difference was found in responses to “If there is a lump in the breast, it is cancer,” where men more frequently agreed with this misconception (5.88%) compared to women (4.46%) (*p* < 0.001). This indicates that men may have a higher rate of misunderstanding regarding the nature of breast lumps and cancer diagnosis. Additionally, a significant gender difference was observed in responses to “Even if there is no family history of breast cancer, a person can still get breast cancer,” with men more likely to incorrectly disagree (4.81%) compared to women (3.72%) (*p* = 0.105), reflecting some misunderstanding of genetic risk factors among male participants (Supplementary Table 6).

### 3.11 What do men think?

Supplementary Table 7 presents the attitudes of male participants toward their spouse or partner receiving a MMG. Among 187 men, 87.8% (*n* = 164) supported their partner’s screening, 4.2% (*n* = 8) did not support it, and 8% (*n* = 15) provided no comment. Among those who supported it, 69.5% (*n* = 114) allowed their partner to go to the doctor, 49.4% (*n* = 81) reminded her of the appointment time, and 68.9% (*n* = 113) encouraged her to attend. Additionally, 45.7% (*n* = 75) scheduled the appointment on her behalf, 68.3% (*n* = 112) accompanied her to the appointment, and 54.3% (*n* = 89) provided financial support. For the eight men who did not support screening, all (100%) believed the appointment would not be helpful, 25% (*n* = 2) cited conflicts with traditions and customs, and 12.5% each (*n* = 1) felt their partner was not at risk of cancer or had no health issues.

## 4 Discussion

This study evaluated BC awareness among a cohort of women and men across various sociocultural strata to identify reasons for low screening participation among women aged 40 years and older. Furthermore, based on findings from all collected data, we aimed to establish a foundation for improving screening uptake.

Genetic and non-genetic risk factors influence the development of BC (19), and female sex is one of the most significant risk factors (20). In our survey, nearly half of the participants (46.47%) correctly identified female gender as a risk factor. More than 50% of both female and male participants were aware that BC may not be limited to women. Despite general awareness, significant gaps remain regarding recognizing established BC risk factors. While a majority (81.6%) of participants acknowledged family history as a potential risk, recognition of other modifiable risk factors, such as alcohol consumption (22.12%), obesity (19.33%), and physical inactivity (17.10%), was relatively low compared to similar studies conducted in Türkiye and Pakistan (13, 21). These three modifiable risk factors, which are among the risk factors for nearly all cancers, should be emphasized as contributing factors in the development of BC, and greater focus should be placed on these issues in awareness campaigns.

Participants showed moderate awareness of major BC symptoms. The most commonly recognized symptoms were palpable breast lumps (81.2%) and axillary masses (65.24%), while awareness of less common signs, such as “peau d’orange” (24.94%), remained low. These results are more favorable compared to two studies reported from Pakistan and similar to the study by Albayrak et al. from Türkiye (13, 21, 22). Overall, men demonstrated a moderate level of knowledge regarding BC symptoms, with the lowest awareness observed for skin changes and nipple discharge. Although 87.8% of men supported their partners’ screening, some expressed concerns, likely due to cultural habits or a lack of information. If overlooked, these attitudes could indirectly influence women’s screening behaviors through family or raising awareness of key BC symptoms. The incidence of male BC is low, but men’s awareness of potential symptoms may encourage them to see a doctor earlier if they notice any abnormalities. Additionally, this awareness could make them more likely to prompt their partners to consult a physician when symptoms arise. Educating the public about lesser-known signs—such as skin changes—through visual demonstrations may enhance recognition of the disease.

There is a large group of women (90.52%) who are aware of BC, screening, and early diagnosis, but somehow, they neglect and do not take it seriously unless it happens to someone close to them. The absence of symptoms in women (36.4%) and negligence (19.7%) were seen as the most prominent indicators of this. Among women who had undergone MMG, 70.59% reported a family history of BC, indicating that personal or familial risk significantly influences screening uptake (*p* = 0.002). This finding corresponds with literature from other developing countries, such as Saudi Arabia, where women feel at risk; they are more likely to engage in protective behavior (23). To address this, public health campaigns should emphasize that MG is recommended for all women over 40, regardless of family history.

The study revealed a relatively high rate of BSE practice (81.04%) compared with reports from Bangladesh and Jordan (12, 24). However, there was no statistically significant difference in SBE performance between women with pre-university and higher education (*p* = 0.070), suggesting that formal education alone may not be an essential factor in improving SBE. This suggests a need to integrate structured SBE training into community health education programs, regardless of participants’ educational backgrounds.

The results of the logistic regression analysis provide useful insights for improving MMG screening rates. Older women were less likely to undergo screening despite being at higher risk for BC, suggesting that awareness campaigns and screening reminders should specifically target this age group. Women who knew someone in their social circle diagnosed with BC were more than twice as likely to get screened, highlighting the powerful role of personal connections in screening decisions. Encouraging women who have already been screened to share their experience with friends and family may be a simple and effective way to increase participation. Higher health literacy was also independently associated with MMG uptake, underscoring the importance of clear and accessible health information in supporting screening decisions.

Healthcare workers were the most common source of BC information for both screened and unscreened women, suggesting that healthcare professionals play a key role in promoting screening. Training healthcare workers to actively discuss screening during routine visits — particularly with patients who have not yet participated — could meaningfully improve uptake. Family and friends were also an important source of information, especially for unscreened women, further supporting the value of peer-based awareness efforts. Digital platforms, including social media, were widely used by both groups, and targeted public health campaigns online could help address common misconceptions and motivate women to get screened.

Beyond individual factors, 17.14% of women reported difficulty accessing healthcare facilities as a barrier to screening, despite free services being available through “KETEM.” Strategies such as mobile MMG units, extended clinic hours, and community outreach programs could help overcome these logistical barriers, particularly for women in rural or underserved areas. Taken together, these findings suggest that improving MMG rates requires a multi-level approach addressing personal, social, educational, and structural factors.

One of the main strengths of this study is its inclusion of both male and female participants, providing a broader perspective on BC awareness and screening attitudes. This inclusive approach allows for the evaluation not only of women’s knowledge and behaviors but also of how men’s awareness and support influence screening participation.

This study has several limitations that should be acknowledged. The cross-sectional design limits causal inference; all associations reported should be interpreted accordingly. Data collection was conducted at a single university hospital and its affiliated institutions in Istanbul, and participants were recruited from pre-selected locations, which may introduce selection bias. Although the sample includes patients and companions across a wide socioeconomic range and despite successfully reaching a demographically diverse population with a wide range of educational and socioeconomic backgrounds, the urban, hospital-based setting may still limit the generalizability of the findings to the broader Turkish population, particularly individuals residing in rural areas or regions with limited healthcare access. The use of self-reported questionnaires introduces the possibility of social desirability bias, particularly regarding screening behaviors and SBE practices. Future research could benefit from a multicenter design with a larger, more representative sample spanning diverse geographic regions and healthcare settings, thereby improving generalizability and providing a more comprehensive understanding of national screening behavior and awareness.

## 5 Conclusion

A considerable proportion of women in our study group appeared to disregard the importance of screening unless a close friend or family member had been affected. Most participants reported seeking medical attention only after symptom onset, which significantly contributes to delayed diagnosis and the resulting treatment challenges. This trend was observed regardless of participants’ education level or health literacy, suggesting well-established cultural or behavioral barriers. Structured and nationwide awareness campaigns led by healthcare professionals are needed to address this issue. Enhancing health literacy and promoting preventive screening behaviors should be emphasized. Addressing education, digital engagement, access to healthcare, screening rates, peer support, and BC awareness can improve these areas, leading to earlier detection and better health outcomes for women.

## Data Availability

The raw data supporting the conclusions of this article will be made available by the authors, without undue reservation.
