# Supplementary material for: Women’s and men’s perspectives on breast cancer in Türkiye: strategies to improve screening participation—a cross-sectional study
Source: Front Public Health. 2026 Jun 24;14:1879455. doi: 10.3389/fpubh.2026.1879455 (PMC13341708; doi:10.3389/fpubh.2026.1879455)

**Supplementary Questionnare-1:**

**BREAST CANCER AWARENESS SURVEY (WOMEN)**

**DEMOGRAPHIC INFORMATION FORM**

**Age**: ...................................................................................

**Marital Status**:
☐ Single - not in a relationship
☐ Single - in a long-term relationship
☐ Married
☐ Divorced
☐ Widowed

**Education Level**:
☐ Primary School
☐ Middle School
☐ High School
☐ University
☐ Literate but never attended school
☐ Other (please specify): .....................................

**Employment Status**:
☐ Unemployed, looking for work
☐ Unemployed, not looking for work
☐ Employed full-time
☐ Employed part-time
☐ Student
☐ Retired
☐ Other: ....................................

**What is your job ?**
(Please write exactly): ...................................................................

**Health Insurance**:
☐ Only public insurance (SGK)
☐ Only private insurance
☐ Both SGK and private insurance
☐ None

**Monthly Income**:
☐ No income
☐ Below minimum wage
☐ Above minimum wage

**Have you ever been diagnosed with a benign breast disease?**
(e.g., cyst, lump, fibroadenoma)
☐ Yes
☐ No

**Do any of your family members or relatives have a history of breast cancer?**
☐ Yes
☐ No
If yes, who? .....................................

**Do you know anyone in your social circle diagnosed with breast cancer?**
☐ Yes
☐ No
If yes, who? .....................................

**Do you have a chronic condition requiring regular doctor visits?**
☐ Yes
☐ No
If yes, please specify: ..................................

**Do you undergo annual health check-ups?**
☐ Yes
☐ No

**HEALTH LITERACY SHORT FORM**

Please indicate how difficult or easy the following tasks are for you:

|  | Very Difficult | Quite Difficult | Quite Easy | Very Easy |
| --- | --- | --- | --- | --- |
| Finding information about the treatment of diseases relevant to you |  |  |  |  |
| Understanding drug leaflets |  |  |  |  |
| Assessing pros and cons of different treatment options |  |  |  |  |
| Calling an ambulance in an emergency |  |  |  |  |
| Finding information on managing mental health issues (e.g., stress, depression) |  |  |  |  |
| Understanding the importance of screening tests (e.g., breast exams, blood sugar tests, blood pressure) |  |  |  |  |
| Deciding which vaccines you might need |  |  |  |  |
| Making decisions based on advice from friends/family about disease prevention |  |  |  |  |
| Finding information about activities that improve mental health (e.g., meditation, walking, exercise) |  |  |  |  |
| Understanding health-related content in media (e.g., internet, newspaper, magazine) |  |  |  |  |
| Understanding how daily habits (eating, drinking, exercise) affect health |  |  |  |  |
| Participating in a sports club or physical activity |  |  |  |  |
| Understanding health conditions like hypertension, diabetes, kidney disease, etc. |  |  |  |  |

**KNOWLEDGE AND PRACTICES ABOUT BREAST CANCER**

**Which of the following are signs of breast cancer?** (You may choose more than one)
☐ Lump in the breast
☐ Peau d’orange (orange peel appearance)
☐ Swelling in the breast
☐ Breast pain
☐ Bloody nipple discharge
☐ Redness/scaling of the breast skin
☐ Nipple retraction
☐ Lump in the armpit
☐ Enlargement of one breast
☐ None of these are signs
☐ Don’t know

**Which of the following increase the risk of breast cancer?** (Choose all that apply)
☐ Performing breast self-exams
☐ Being female
☐ Being overweight
☐ Using birth control pills
☐ Aging
☐ Having a family history of breast cancer
☐ Never having given birth
☐ Using hormone replacement therapy after menopause
☐ Alcohol consumption
☐ Physical inactivity
☐ Never breastfeeding
☐ None of these are risk factors
☐ Don’t know

**Do you perform breast self-examinations?**
☐ Yes
☐ No
☐ I don’t know what a breast self-exam is

**If yes, how often do you perform it?**
☐ Daily
☐ Weekly
☐ Monthly
☐ Every 6 months
☐ Occasionally
☐ Other: ...........

**BREAST CANCER SCREENING AWARENESS**

**Do you know that breast cancer can be detected early with mammography?**
☐ Yes
☐ No

**At what age should mammography screening begin?**
☐ Before age 40
☐ Age 40
☐ Age 50
☐ After age 60
☐ Don’t know

**How often should mammograms be done?**
☐ Every year
☐ Every 2 years
☐ Only when symptoms appear
☐ Don’t know

**Have you ever had a mammogram?**
☐ Yes
☐ No

**If yes, where?** (You may choose more than one)
☐ KETEM (Early Cancer Detection Center)
☐ Public Hospital
☐ Private Hospital
☐ Private Imaging Center/Clinic
☐ Other: ………………

**If yes, how frequently?**
☐ Once a year
☐ Every 2 years
☐ Irregularly

**Why don’t you have regular breast checks?** (Select all that apply)
☐ No complaints about my breasts
☐ I don’t believe I’m at risk
☐ Forgetfulness/Negligence
☐ Lack of time
☐ My partner doesn’t want me to
☐ No one in my community gets it done
☐ Fear of mammography
☐ Fear of finding something
☐ Difficulty accessing healthcare
☐ Fear of hospitals
☐ Embarrassment about showing breasts
☐ I don’t believe mammography works
☐ I'm not at screening age
☐ Don’t know where to make an appointment
☐ Can’t get an appointment
☐ Transportation difficulties
☐ Financial constraints
☐ Other: ………………

**Where do you get your information about breast cancer and screening?** (Select all that apply)
☐ Family/Friends
☐ Healthcare workers
☐ TV/Radio
☐ Books
☐ Newspapers
☐ Internet (Google)
☐ Facebook
☐ Twitter
☐ YouTube
☐ Instagram
☐ Other: ………………

**TRUE / FALSE / I DON’T KNOW STATEMENTS**

Please indicate whether you think the following statements are **True**, **False**, or **Don’t Know**:

|  | **True** | **False** | **Don’t Know** |
| --- | --- | --- | --- |
| Breast cancer is a rare disease |  |  |  |
| Only women get breast cancer |  |  |  |
| Breast cancer does not occur in young women |  |  |  |
| A lump in the breast always means cancer |  |  |  |
| A person can get breast cancer even without a family history |  |  |  |
| A biopsy does not cause cancer to spread |  |  |  |
| Breast cancer can be completely cured if detected early |  |  |  |

**Supplementary Questionnare-2:**

**BREAST CANCER AWARENESS SURVEY (MEN)**

**DEMOGRAPHIC INFORMATION FORM**

**Age**: ............................................................................................

**Marital Status**:
☐ Single – not in a relationship
☐ Single – in a long-term relationship
☐ Married
☐ Divorced
☐ Widowed

**Education Level**:
☐ Primary School
☐ Middle School
☐ High School
☐ University
☐ Literate but never attended school
☐ Other (please specify): .....................................

**Employment Status**:
☐ Unemployed, seeking work
☐ Unemployed, not seeking work
☐ Full-time employed
☐ Part-time employed
☐ Student
☐ Retired
☐ Other: ....................................

**What is your job and in which sector?** (Please specify): .............................................................

**Health Insurance**:
☐ Public insurance only (SGK)
☐ Private insurance only
☐ Both SGK and private
☐ None

**Monthly Income**:
☐ No income
☐ Below minimum wage
☐ Above minimum wage

**Do you have a family member/relative diagnosed with breast cancer?**
☐ Yes
☐ No
If yes, who? ...........................

**Do you know anyone in your social circle diagnosed with breast cancer?**
☐ Yes
☐ No
If yes, who? ...........................

**Do you have a condition requiring regular doctor visits?**
☐ Yes
☐ No
If yes, please specify: ....................

**Do you get annual health screenings?**
☐ Yes
☐ No

**HEALTH LITERACY SHORT FORM**

Please indicate how difficult or easy the following tasks are for you:

|  | **Very Difficult** | **Quite Difficult** | **Quite Easy** | **Very Easy** |
| --- | --- | --- | --- | --- |
| Finding information about the treatment of diseases relevant to you |  |  |  |  |
| Understanding drug leaflets |  |  |  |  |
| Assessing pros and cons of different treatment options |  |  |  |  |
| Calling an ambulance in an emergency |  |  |  |  |
| Finding information on managing mental health issues (e.g., stress, depression) |  |  |  |  |
| Understanding the importance of screening tests (e.g., breast exams, blood sugar tests, blood pressure) |  |  |  |  |
| Deciding which vaccines you might need |  |  |  |  |
| Making decisions based on advice from friends/family about disease prevention |  |  |  |  |
| Finding information about activities that improve mental health (e.g., meditation, walking, exercise) |  |  |  |  |
| Understanding health-related content in media (e.g., internet, newspaper, magazine) |  |  |  |  |
| Understanding how daily habits (eating, drinking, exercise) affect health |  |  |  |  |
| Participating in a sports club or physical activity |  |  |  |  |
| Understanding health conditions like hypertension, diabetes, kidney disease, etc. |  |  |  |  |

**ATTITUDE TOWARDS BREAST CANCER SCREENING**

**Do you support your partner getting a mammogram?**
☐ YES (If yes, please indicate how you support them – you may select multiple)
☐ I allow her to go to the doctor
☐ I remind her of the doctor’s appointment
☐ I encourage her to go
☐ I make the appointment
☐ I go with her
☐ I provide financial support
☐ Other: .................

☐ NO (If no, please indicate why – you may select multiple)
☐ I don’t believe it helps (if it’s going to happen, it will...)
☐ It’s against our traditions/customs
☐ We have financial difficulties
☐ I don’t believe my wife/partner will get cancer
☐ My wife/partner has no complaints
☐ She is too young for a mammogram
☐ Other: .................

☐ I leave this decision to my wife/partner
☐ I don’t have any knowledge on this subject

**Where do you get information about breast cancer and screening?** (Select all that apply)
☐ Family/Friends
☐ Health professionals
☐ TV/Radio
☐ Books
☐ Newspapers
☐ Internet (Google)
☐ Facebook
☐ Twitter
☐ YouTube
☐ Instagram
☐ Other: .................

**BREAST CANCER AWARENESS QUESTIONS**

**Which of the following are signs of breast cancer?** (Circle all that apply)

- Lump in the breast
- Peau d’orange (orange peel appearance)
- Swelling in the breast
- Pain in the breast
- Bloody nipple discharge
- Redness/scaling of breast skin
- Nipple retraction
- Lump in the armpit
- Enlargement of the breast
- None of these
- Don’t know

**TRUE / FALSE / DON’T KNOW STATEMENTS**

Please indicate whether you think the following statements are **True**, **False**, or **Don’t Know**:

|  | **True** | **False** | **Don’t Know** |
| --- | --- | --- | --- |
| Breast cancer is a rare disease |  |  |  |
| Only women get breast cancer |  |  |  |
| Breast cancer does not occur in young women |  |  |  |
| A lump in the breast always means cancer |  |  |  |
| A person can get breast cancer even without a family history |  |  |  |
| A biopsy does not cause cancer to spread |  |  |  |
| Breast cancer can be completely cured if detected early |  |  |  |

**Supplementary Table-1 : Health Literacy of the participants**

|  | | **Total** | **p-value** | **Women (n: 407)** | **p-value** | **Men (n: 187)** | **p-value** |
| --- | --- | --- | --- | --- | --- | --- | --- |
| **Level of Education** | **Undergraduate** | 32.949  ± 9.862 | **0.002** | 32.575  ± 9.265 | **<0.001** | 33.560  ±10.792 | 0.481 |
|  | **Postgraduate** | 35.433  ± 8.659 |  | 35.756  ± 8.762 |  | 34.557  ± 8.359 |  |
| **Monthly Income** | **Below Minimum Wage** | 33.477 ± 9.918 | 0.130 | 34.041  ± 9.444 | 0.400 | 30.339  ± 11. 966 | **0.038** |
|  | **Above Minimum Wage** | 34.764  ± 8.966 |  | 34.841  ± 8.881 |  | 34.635 ± 9.131 |  |

*Health literacy scores are presented as mean ± standard deviation. Comparisons between education levels and income groups were performed using the independent samples t-test.*

*A p-value ≤ 0.05 was considered statistically significant*

**Supplementary Table 2: Women Participants' knowledge of breast cancer risk factors**

|  | | Women (n:538) | Level of education | | | Monthly income | | |
| --- | --- | --- | --- | --- | --- | --- | --- | --- |
|  | |  | **Under**  **graduate** | **Post**  **graduate** | **p-value** | **Below wage** | **Above wage** | **p-value** |
| Being women | **Yes** | 288 (46.47%) | 114 (39.58%) | 174 (60.42%) | **0.050** | 90 (31.25%) | 198 (68.75%) | 0.930 |
|  | **No** | 250 (53.53%) | 120 (48%) | 130 (52%) |  | 79 (31.60%) | 171 (68.40%) |  |
| Family history of breast cancer | **Yes** | 439 (81.60%) | 172 (39.18%) | 267 (60.82%) | **<0.001** | 128 (29.16%) | 311 (70.84%) | **0.018** |
|  | **No** | 99 (18.40%) | 62 (62.63%) | 37 (37.37%) |  | 41 (41.41%) | 58 (58.59%) |  |
| Alcohol consumption | **Yes** | 119 (22.12%) | 51 (42.86%) | 68 (57.14%) | 0.874 | 43 (36.13%) | 76 (63.87%) | 0.209 |
|  | **No** | 419 (77.88%) | 183 (43.68%) | 236 (56.32%) |  | 126 (30.07%) | 293 (69.93%) |  |
| Obesity | **Yes** | 104 (19.33%) | 46 (44.23%) | 58 (55.77%) | 0.866 | 30 (28.85%) | 74 (71.15%) | 0.530 |
|  | **No** | 434 (80.67%) | 188 (43.32%) | 246 (56.68%) |  | 139 (32.03%) | 295 (67.97%) |  |
| Having never given birth | **Yes** | 103 (19.14%) | 40 (38.83%) | 63 (61.17%) | 0.289 | 26 (25.24%) | 77 (74.76%) | 0.134 |
|  | **No** | 435 (80.86%) | 194 (44.60%) | 241 (55.40%) |  | 143 (32.87%) | 292 (67.13%) |  |
| Being not physically active | **Yes** | 92 (17.10%) | 30 (32.61%) | 62 (67.39%) | **0.021** | 26 (28.26%) | 66 (71.74%) | 0.474 |
|  | **No** | 446 (82.90%) | 204 (45.74%) | 242 (54.26%) |  | 143 (32.06%) | 303 (67.94%) |  |
| Use oral contraceptive pills | **Yes** | 108 (20.07%) | 38 (35.19%) | 70 (64.81%) | **0.051** | 34 (31.48%) | 74 (68.52%) | 0.986 |
|  | **No** | 430 (79.93%) | 196 (45.58%) | 234 (54.42%) |  | 135 (31.40%) | 295 (68.60%) |  |
| Hormone replacement therapy after menopause | **Yes** | 146 (27.14%) | 60 (41.10%) | 86 (58.90%) | 0.493 | 50 (34.25%) | 96 (65.75%) | 0.387 |
|  | **No** | 392 (72.86%) | 174 (44.39%) | 218 (55.61%) |  | 119 (30.36%) | 273 (69.64%) |  |
| Lack of breastfeeding | **Yes** | 119 (22.12%) | 46 (38.66%) | 73 (61.34%) | 0.228 | 30 (25.21%) | 89 (74.79%) | 0.099 |
|  | **No** | 419 (77.88%) | 188 (44.87%) | 231 (55.13%) |  | 139 (33.17%) | 280 (66.83%) |  |
| Aging | **Yes** | 67 (12.45%) | 18 (26.87%) | 49 (73.13%) | **0.003** | 20 (29.85%) | 47 (70.15%) | 0.768 |
|  | **No** | 471 (87.55%) | 216 (45.86%) | 255 (54.14%) |  | 149 (31.63%) | 322 (68.37%) |  |

*Statistical comparisons between education levels and monthly income groups were performed using the chi-square test. A p-value ≤ 0.05 was considered statistically significant.*

**Supplementary Table 3: Participants' knowledge of breast cancer screening (women)**

|  | | Women (n:538) | Level of Education | | | Monthly Income | | |
| --- | --- | --- | --- | --- | --- | --- | --- | --- |
|  | |  | **Pre-university** | **Higher education** | **p-value** | **Below minimum wage** | **Above minimum wage** | **p-value** |
| Are you aware that breast cancer can be detected early through mammography screening? | **Yes** | 487 (90.52%) | 207 (42.51%) | 280 (57.49%) | 0.153 | 145 (29.77%) | 342 (70.23%) | 0.011 |
|  | **No** | 51 (9.48%) | 27 (52.94%) | 24 (47.06%) |  | 24 (47.06%) | 27 (52.94%) |  |
| At what age do you think breast cancer screening with mammography should begin? | **Yes (initiation at 40)** | 241 (44.80%) | 98 (40.66%) | 143 (59,34%) | 0.285 | 60 (24.90%) | 181 (75.10%) | **0.001** |
|  | **No (not at 40)** | 260 (48.33%) | 122 (46.92%) | 138 (53.08%) |  | 89 (34.23%) | 171 (65.77%) |  |
|  | **No comment** | 37 (6.88%) | 14 (37,84%) | 23 (62.16%) |  | 20 (54.05%) | 17 (45.95%) |  |
| How often do you think mammography should be performed? | **Yes (every year)** | 311 (57,81%) | 132 (42.44%) | 179 (57.56%) | 0.592 | 89 (28.62%) | 222 (71.38%) | **0.045** |
|  | **No (not every year)** | 172 (31,97%) | 80 (46.51%) | 92 (53.49%) |  | 55 (31.98%) | 117 (68.02%) |  |
|  | **No comment** | 55 (10,22%) | 22 (40%) | 33 (60%) |  | 25 (45.45%) | 30 (54.55%) |  |

*Statistical comparisons between education levels and monthly income groups were performed using the chi-square test.*

*A p-value ≤ 0.05 was considered statistically significant.*

**Supplementery Table 4: Sources of Breast Cancer Awareness Among Women Who Have and Have Not Performed Mammography Aged 40 and Above**

**(Participants were allowed to select more than one source)**

|  | Performed Mammography (n: 204) | Not Performed Mammography (n:89) |
| --- | --- | --- |
| Family & friends | 91 (44,61%) | 45 (50,56%) |
| Healthcare worker | 136 (66,67%) | 49 (55,06%) |
| TV/Radio | 70 (34,31%) | 32 (35,95%) |
| Books | 19 (9,31%) | 7 (7,86%) |
| Newspaper | 21 (10,29%) | 6 (6,74%) |
| Internet | 92 (45,10%) | 44 (49,44%) |
| Facebook | 17 (8,33%) | 5 (5,62%) |
| Twitter | 12 (5,88%) | 2 (2,25%) |
| Youtube | 24 (11,76%) | 13 (14,61%) |
| Instagram | 32 (15,69%) | 15 (16,85%) |

**Supplementary Table 5: Participants' knowledge of self-breast examination**

|  | | Women (n:538) | Level of education | | |
| --- | --- | --- | --- | --- | --- |
|  | |  | **Pre-university** | **Higher education** | **p-value** |
| Self-breast examination habit | **Yes** | 436 (81,04%) | 200 (45.87%) | 236 (54.13%) | 0.070 |
|  | **No** | 71 (13,20%) | 24 (33.80%) | 47 (66.20%) |  |
|  | **No comment** | 31 (5,76%) | 10 (32.26%) | 21 (67.74%) |  |

|  | | Women (n:436) | Level of education | | |
| --- | --- | --- | --- | --- | --- |
| If yes, | |  | **Pre-university** | **Higher education** | **p-value** |
| Self-breast examination habit | **Every day** | 12 (2,75%) | 4 (33.33%) | 8 (66.67%) | **<0.001** |
|  | **Once a week** | 64 (14,68%) | 26 (40.63%) | 38 (59.38%) |  |
|  | **Once a month** | 100 (22,94%) | 37 (37%) | 63 (63%) |  |
|  | **Once in six months** | 23 (5,28%) | 17 (73.91%) | 6 (26.09%) |  |
|  | **Randomly Irregular periods** | 200 (45,87%) | 110 (55%) | 90 (45%) |  |
|  | **Others** | 37 (8,49%) | 6 (16,22%) | 31 (83,78%) |  |

*Statistical comparisons between education levels were performed using the chi-square test. For variables with expected cell frequencies below 5, Fisher's exact test was applied.*

*A p-value ≤ 0.05 was considered statistically significant.*

**Supplementary Table 6: Knowledge about breast cancer**

|  | | **Women** | **Men** | **p-value** | **Women (n: 407)** | | | **Men (n: 187)** | | |
| --- | --- | --- | --- | --- | --- | --- | --- | --- | --- | --- |
|  |  |  |  |  | **Undergraduate** | **Postgraduate** | **p-value** | **Undergraduate** | **Postgraduate** | **p-**  **value** |
| **Breast cancer is a rare disease.** | **Yes** | 24 (5,9%) | 29(15,51%) | **<0.001** | 10 (6.6%) | 14 (5.5%) | 0.459 | 19 (20.4%) | 10 (10.6%) | **0.037** |
|  | **No** | 315(77,4%) | 125(66,84%) |  | 121 (79.6%) | 194 (76.1%) |  | 54 (58.1%) | 71 (75.5%) |  |
|  | **No comment** | 68 (16,7%) | 33 (17,65%) |  | 21 (13.8%) | 47 (18.4%) |  | 20 (21.5%) | 13 (13.8%) |  |
| **Only women get breast cancer.** | **Yes** | 89 (21,87%) | 44 (23,53%) | 0.329 | 43 (28.3%) | 46 (18%) | **0.024** | 26 (28%) | 18 (19.1%) | 0.054 |
|  | **No** | 232 (57%) | 95 (50,80%) |  | 75 (49.3%) | 158 (62%) |  | 39 (41.9%) | 56 (59.6%) |  |
|  | **No comment** | 86 (21,13%) | 48 (25,67%) |  | 34 (22.4%) | 51 (20%) |  | 28 (30.1%) | 20 (21.3%) |  |
| **Breast cancer is not seen in young women.** | **Yes** | 14 (3,44%) | 8 (4,28%) | **0.016** | 8 (5.3%) | 6 (2.4%) | 0.296 | 5 (5.4%) | 3 (3.2%) | 0.227 |
|  | **No** | 342(84,03%) | 139(74,33%) |  | 125 (82.2%) | 217 (85.1%) |  | 64 (68.8%) | 75 (79.8%) |  |
|  | **No comment** | 51 (12,54%) | 40 (21,39%) |  | 19 (12.5%) | 32 (12.5%) |  | 24 (25.8%) | 16 (17%) |  |
| **If there is a lump in the breast, it is cancer.** | **Yes** | 18 (4,42%) | 11 (5,88%) | **0.002** | 11 (7.2%) | 7 (2.7%) | **<0.001** | 9 (9.7%) | 2 (2.1%) | **0.027** |
|  | **No** | 314(77,15%) | 119(63,64%) |  | 102 (67.1%) | 212 (83.1%) |  | 52 (55.9%) | 67 (71.3%) |  |
|  | **No comment** | 75 (18,43%) | 57 (30,48%) |  | 39 (25.7%) | 36 (14.1%) |  | 32 (34.4%) | 25 (26.6%) |  |
| **Even if there is no family history of breast cancer, a person can still get breast cancer.** | **Yes** | 359(88,21%) | 154(82,35%) | 0.155 | 130 (85.5%) | 229 (89.8%) | 0.339 | 70 (75.3%) | 84 (89.4%) | **0.040** |
|  | **No** | 13 (3,19%) | 9 (4,81%) |  | 7 (4.6%) | 6 (2.4%) |  | 6 (6.5%) | 3 (3.2%) |  |
|  | **No comment** | 35 (8,6%) | 24 (12,83%) |  | 15 (9.9%) | 20 (7.8%) |  | 17 (18.3%) | 7 (7.4%) |  |
| **Having a biopsy does not cause a lump in the breast to become cancerous.** | **Yes** | 207(50,86%) | 169(90,37%) | **<0.001** | 80 (52.6%) | 127 (49.8%) | 0.857 | 81 (87.1%) | 88 (93.6%) | 0.194 |
|  | **No** | 31 (7,62%) | 2 (1,07%) |  | 11(7.2%) | 20 (7.8%) |  | 2 (2.2%) | 0 (0%) |  |
|  | **No comment** | 169(41,52%) | 16 (8,56%) |  | 61(40.1%) | 108 (42.4%) |  | 10 (10.8%) | 6 (6.4%) |  |
| **Breast cancer can be completely treated with early diagnosis.** | **Yes** | 332(81,57%) | 145(77,54%) | 0.387 | 131 (86.2%) | 201 (78.8%) | 0.173 | 76 (81.7%) | 69 (73.4%) | 0.312 |
|  | **No** | 16 (3,93%) | 7 (3,74%) |  | 4 (2.6%) | 12 (4.7%) |  | 2 (2.2%) | 5 (5.3%) |  |
|  | **No comment** | 59 (14,50%) | 35 (18,72%) |  | 17 (11.2%) | 42 (16.5%) |  | 15 (16.1%) | 20 (21.3%) |  |

**Supplementary Table 7: Male participants’ attitudes toward breast cancer screening**

|  | | Men (n:187) |
| --- | --- | --- |
| Do you support your partner getting a mammogram? | Yes | 164 (87,8%) |
|  | No | 8 (4,2%) |
|  | No Comment | 15 (8%) |

|  |  |
| --- | --- |
| If yes, (Participants were allowed to select more than one choice) | **Men (n:164)** |
| I allow her to go to doctor. | 114 (69,51%) |
| I encourage her to go to the appointment. | 113 (68,90%) |
| I go to the appointment with her. | 112 (68,29%) |
| I provide financial support. | 89 (54,27%) |
| I remind her of the doctor’s appointment time. | 81 (49,39%) |
| I schedule the doctor appointment for her. | 75 (45,73%) |

**Supplementary Figure 1: Mammography Screening, Education Level, and Family/Social History in Women**

**(Aged 40 and Above)**


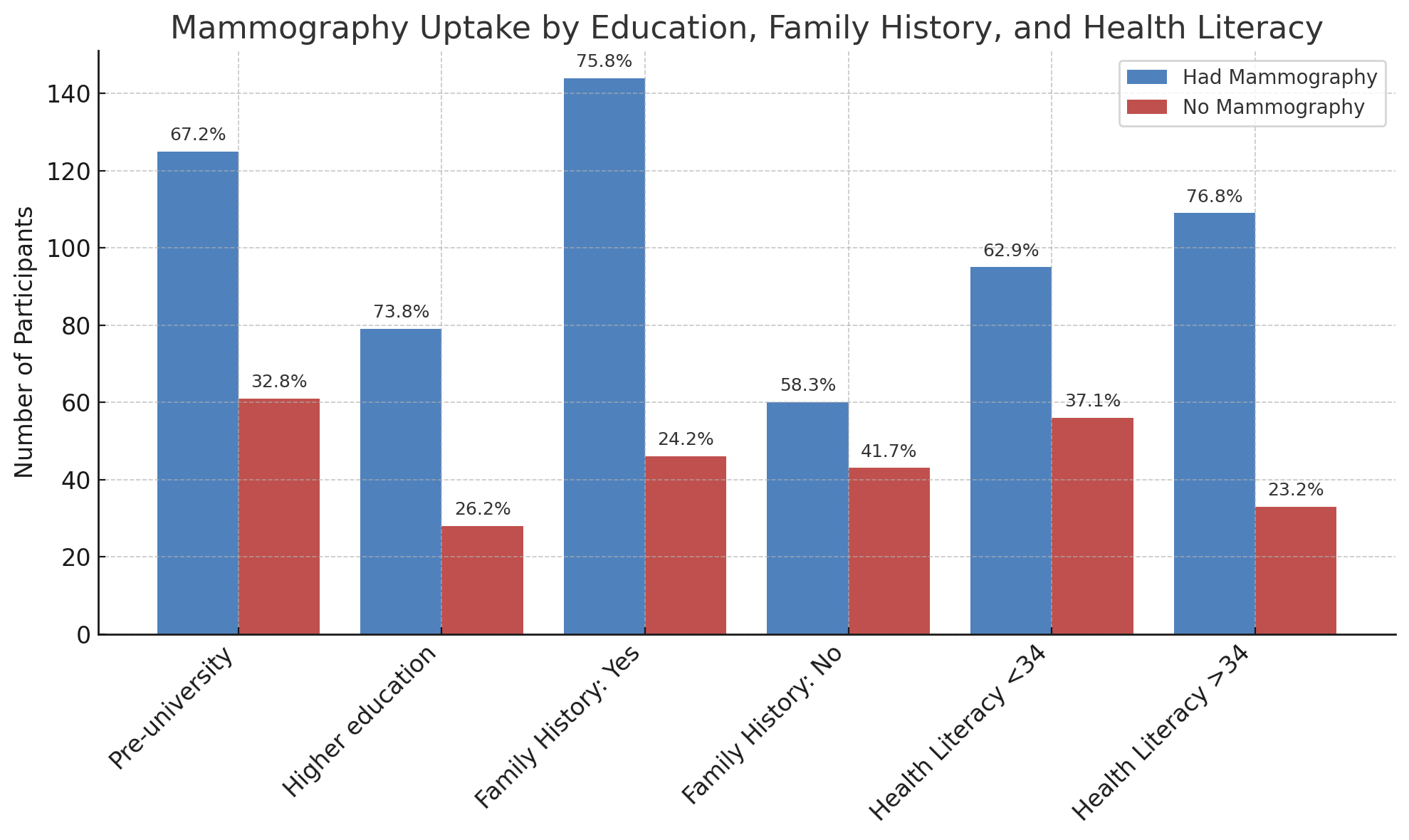


**Supplementary Figure 2: Screening Location and Frequency Patterns among Female Patients who Have Undergone Mammography**


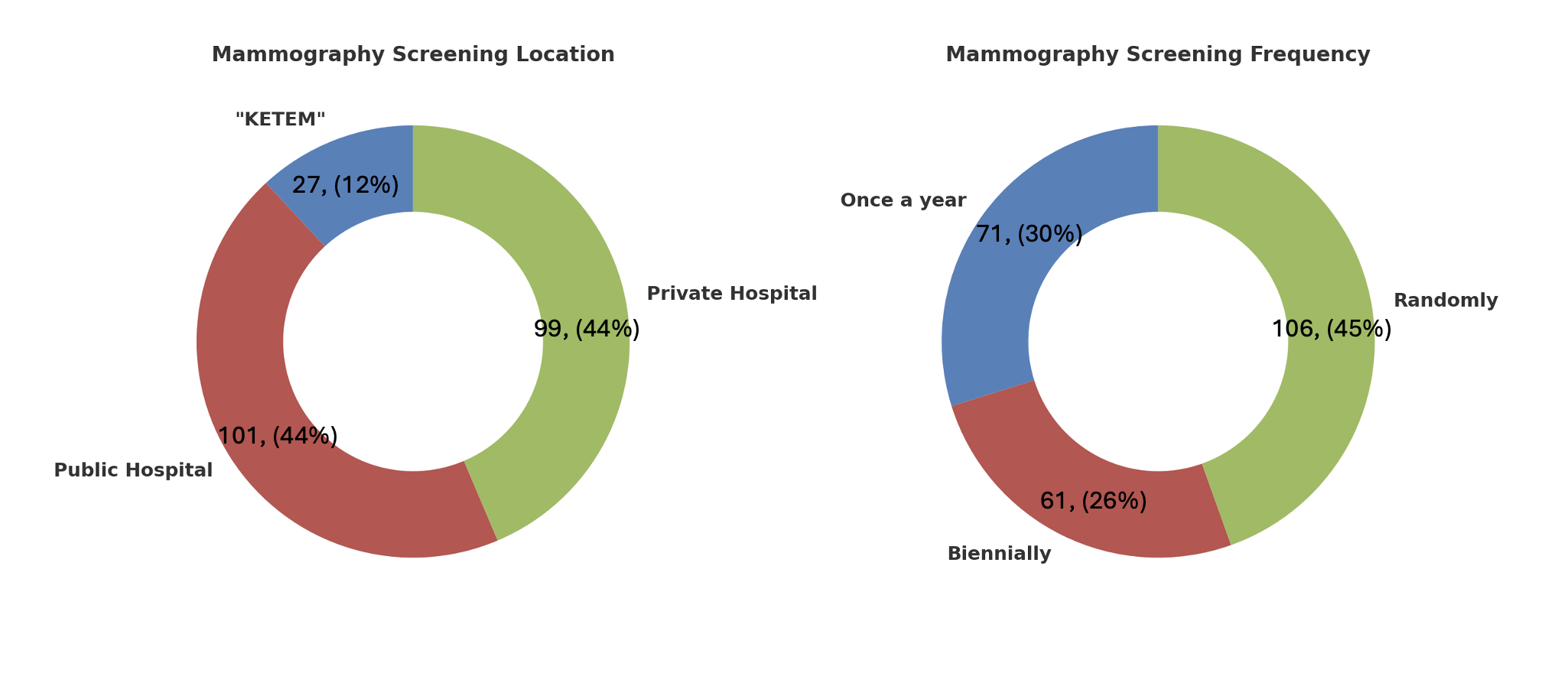

Supplement: Supplementary file 1 [file Data_Sheet_1.docx]
